# Does Adding Silver Nanoparticles to Leukocyte- and Platelet-Rich Fibrin Improve Its Properties?

**DOI:** 10.1155/2018/8515829

**Published:** 2018-05-27

**Authors:** Hooman Khorshidi, Pardis Haddadi, Saeed Raoofi, Parisa Badiee, Ali Dehghani Nazhvani

**Affiliations:** ^1^Department of Periodontology, School of Dentistry, Shiraz University of Medical Sciences, Shiraz, Iran; ^2^Professor Alborzi Clinical Microbiology Research Center, Shiraz University of Medical Sciences, Shiraz, Iran; ^3^Department of Oral & Maxillofacial Pathology, Biomaterial Research Center, School of Dentistry, Shiraz University of Medical Sciences, Shiraz, Iran

## Abstract

**Objectives:**

Leucocyte- and platelet-rich fibrin (L-PRF) membrane can be used in various regenerative treatments. In the case of classical heterologous membrane exposure, microorganisms can be colonized on it and jeopardize the success of treatment. The aim of this study was to compare the antibacterial, mechanical, and histologic characteristics of the L-PRF membrane before and after the addition of silver nanoparticles (SNP).

**Materials and Method:**

This study was performed on 10 volunteer men aged 25-35 years. 20 ml whole bloods were collected from each person and L-PRFs were made by routine and SNP modified method. Mechanical, antibacterial, and histological properties were evaluated.

**Results:**

The antibacterial efficacy of L-PRF and nanosilver-modified L-PRF was presented as* Klebsiella pneumonia* had growth on the L-PRF membrane after 12 hours. After 24 hours,* Klebsiella pneumonia* and* Streptococcus viridans* had growth on L-PRF and only* Klebsiella pneumonia* had growth on SNP-L-PRF. The tensile strength and stiffness were significantly higher in the SNP-L-PRF. Precipitation of the SNPs was patchy in the outer layers and quite homogeneous in the inner core.

**Conclusion:**

Modification of L-PRF with SNP improves the mechanical properties and antibacterial activity of the L-PRF. It can play an important role in regenerative procedures.

## 1. Introduction

Guided tissue regeneration (GTR) and guided bone regeneration (GBR) are surgical techniques that aim to reconstruct the damaged periodontal tissues which are lost due to periodontal lesions and to regain the alveolar bone, lost due to tooth extraction or periodontal disease [1]. These methods employ various membranes to cover the bone and periodontal ligament and temporarily separate them from the epithelium and gingival connective tissue [2].

Regenerative potential of platelets was first introduced in the 1970s, just when they were found to contain growth factors responsible for increasing the collagen production, cell mitosis, blood vessels growth, and induction of cell differentiation [3]. The platelets were increasingly used in tissue regeneration over time. The platelet-rich fibrin can be used in various regenerative treatments to accelerate the healing and improve the regeneration procedure [4]. It can also be used as a scaffold in tissue engineering [5].

Nowadays, there are several techniques to obtain the high concentration of platelets, each of which results in a specific product that is unique in terms of biology and performance. These methods are generally classified into four groups based on their fibrin and leucocyte content: pure platelet-rich plasma (P-PRP), leucocyte- and platelet-rich plasma (L-PRP), pure platelet-rich fibrin (P-PRF), and leucocyte- and platelet-rich fibrin (L-PRF) [6].

The chemical and physical properties of the membrane can influence the ultimate outcome of GBR and GTR [7]. The tensile strength of the tissue or the material which is sutured affects the success of suturing and the clinical results of wound healing [8]. Meanwhile, the membrane stiffness and presence of stiff material influence the distribution of mechanical forces over the surrounding tissues [9]. Generally, the better the mechanical properties of the membrane provide the better support for regenerative treatments.

Among the mentioned methods for the preparation of high platelet concentrations, the L-PRF results in the formation of a strong fibrin matrix which can remodel slowly in the tissue, and if pressed, it turns to a strong membrane with better mechanical properties like tensile strength [6, 10]. In this technique, the patient's venous blood is centrifuged within an anticoagulant-free tube at low speed. Consequently, the L-PRF is formed in the middle between red blood cells in the bottom and plasma layer in the above [11].

The most frequent postoperative complication of different regenerative techniques is the membrane exposure to the oral cavity, in which case, oral cavity microorganisms can colonize on the membrane and jeopardize the success of treatment [12]. It results in higher risk of infection and poor bone healing even in healthy individuals. Reinforcing the membranes' antimicrobial properties with inorganic materials can improve the treatment results. Inorganic antimicrobial materials have been more appreciated recently due to their safety and stability.

One of the substances widely used today in various medical fields is silver nanoparticle (SNP). It has been shown that these particles have high biocompatibility and also have favorable properties, including antimicrobial properties [13]. Studies reported the effect of these materials on a wide spectrum of gram-negative and gram-positive bacteria as well as antibiotic-resistant species. Additionally, their antifungal and antiviral effects were proven [12]. The purpose of this study was to compare the antibacterial, mechanical, and histologic characteristics of the L-PRF membrane before and after the addition of SNPs.

## 2. Materials and Method

This study was performed on 10 volunteers selected on the basis of their availability. The inclusion criteria were male sex and age range of 25-35 years. The exclusion criteria were the history of the known systemic diseases, history of anticoagulant drugs assumption, smoking, use of any medication within the last three months, and lack of satisfaction.

### 2.1. Preparing SNP Suspension

To obtain a uniform suspension, 0.1 gr nanosilver powder with particles sized <100 nm (Sigma Aldrich; USA), along with 1 cc normal saline, was poured into the tube and sonicated at 200 W for 2 minutes in a sonicator device [14].

### 2.2. Blood Samples Collection

In this experimental study, after obtaining informed consent approved by the Ethics Committee of Shiraz University of Medical Sciences (code# 14866), 19 ml venous blood was taken from the subjects. Then, 10 ml of the obtained blood sample was poured into a dry sterile tube and the remaining 9 ml in addition to 1 ml of SNP suspension was poured into another tube and gently shook with hand to achieve a uniform 1% concentration.

### 2.3. Making L-PRF and SNP Modified L-PRF

The previously mentioned tubes were centrifuged at 2700 rpm for 12 minutes to produce original L-PRF (Intra-Spin, Intra-Lock); the only CE and FDA cleared system for the preparation of L-PRF [15]. The fibrin clot containing platelets was formed in the middle of the tubes, with the red blood cells lying at the bottom and the plasma on the top. The fibrin clots were removed from the tubes and separated from the underlying layers. The clots were placed on the metal grid in XPression Box (Intra-Lock). The metal lid was placed over the samples for 10 minutes to form them under pressure. They were used to evaluate the antimicrobial properties.

Prior to evaluation of the mechanical properties, the samples needed to be equalized in terms of shape and size. Thus, a plexiglass mold was prepared as inspired by Alston's method [16]. The mold was dog-bone-shaped of 2-mm thickness all over and 2-mm width in the middle. This part was the weakest area for stress accumulation and rupture.

### 2.4. Evaluation of Antimicrobial Characteristics

1 ml unstimulated saliva was collected in sterile tube from three volunteers and mixed for 3ml saliva pool. They were requested to avoid eating and drinking within the two preceding hours. To evaluate the microflora of saliva pool, it was cultured on blood agar (Liofilchem; Italy) and Thioglycolate broth (Merck; Germany). Each fibrin membrane was divided into smaller pieces and incubated with 1 ml of saliva pool at 37°C for 24 hours.

After incubation, each piece was rinsed three times with sterile normal saline, placed on the shaker for 30 seconds, and centrifuged for 1 minute to remove the surrounding saliva, so as to solely evaluate the microbial biofilm formed on the sample. Then, 500 *μ*l of the RPMI 1640 medium (Sigma; St. Louis, Missouri) was poured over the samples and incubated at 37°C for 12 and 24 hours. After incubation periods, 200 *μ*l of media was poured in 96 well plates and evaluated by Elisa Reader device (Thermolabsystem, Multiskan Ascent, Finland) at 578 nm. Positive and negative controls in this study were the saliva cultured in RPMI and RPMI medium, respectively [17]. The isolated microorganisms were identified by using API 20 E (BioMerieux), Optochin test, and biliary solution.

### 2.5. Evaluation of the Mechanical Properties

The tensile test was done by using the Universal testing machine (Zwick/Roell; 2020, Germany). The sample was fixed in the device. The tensile force was applied at 2mm/min speed, while the stress-strain curve was being simultaneously drawn by the Test Expert II software. The force was applied until the sample was torn from the thin middle part. The test finished as the sample ruptured. Then, the curve was used to measure the samples' tensile strength, toughness, and stiffness.

### 2.6. Evaluation of Histological Properties

The remaining pieces of the membranes were fixed in 10% formalin for 24 hours to be subjected to H&E staining and evaluated with the light microscope.

### 2.7. Statistical Data Analysis

The obtained data was entered into Microsoft Excel software to find the area under the curve (toughness) and the slope (stiffness). The tensile strength of the samples was calculated by dividing the maximum force leading to membrane rupture by their cross-section area (4 mm^2^). Paired* t*-test was used to compare these values between the two types of membranes and* P* < 0.05 was considered to be statistically significant.

## 3. Results

### 3.1. Antimicrobial Properties

The* Streptococcus viridans *and* Klebsiella pneumoniae* species were isolated from the primary saliva pool culture. After 12 hrs incubation of membrane in RPMI 1640, the turbidity (absorbance) of medium (negative control) at 578 nm wavelength was equal to the SNP modified L-PRF membrane, whereas in the L-PRF membrane the turbidity was higher which indicated the growth of some microorganisms. Identification of the isolated species indicated the growth of* K. pneumoniae* on the L-PRF membrane after 12 hours. After 24 hours, the SNP modified L-PRF revealed the presence of* K. pneumonia*, while the L-PRF showed both* K. pneumoniae* and* S. viridans.*

### 3.2. Mechanical Properties

#### 3.2.1. Tensile Strength

The mean tensile strength was 0.154±0.05 MPa in L-PRF membrane samples and 0.435±0.19 MPa in L-PRF samples modified by SNPs, being statistically significantly higher in the latter (*P* = 0.01).

#### 3.2.2. Stiffness

The mean stiffness value was 0.009 ± 0.004 MPa in L-PRF and 0.05 ± 0.02 MPa in L-PRF membranes modified by SNPs, being significantly higher in the latter (*P* = 0.01).

#### 3.2.3. Toughness

The mean toughness was measured to be 63.8 ± 22.4 J/mm^3^ in L-PRF and 74.29 ± 35.1 J/mm^3^ in SNP modified L-PRF. Although the toughness was higher in nanosilver group, the difference was not statistically significant (*P* = 0.4)

### 3.3. Histological Properties

**Figure 1:**
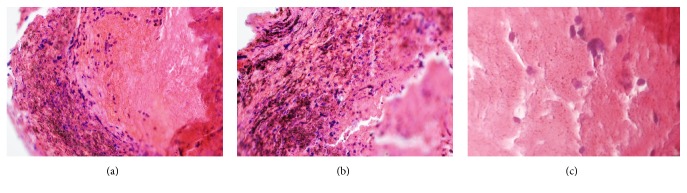
Microscopic sections show silver nanoparticles all over the membrane ((a), 100X); in outer layers they were more densely seated in the fibrin meshwork ((b), 200X) compared with the inner layers. Precipitation of the AgNPs was patchy in outer layers and quite homogeneous in inner layers ((c), 400X). Leukocytes were also denser in outer layers than inner layers (H&E staining).

Evaluating the microscopic sections, the silver nanoparticles were observed all over the membrane, but in the outer layers they were more densely attached to the fibrin strands compared with the inner layers. Precipitation of the SNPs was patchy in the outer layers and quite homogeneous in the inner layers. Moreover, the leukocytes were denser in the outer layers than in the inner layers (Figures 1(a), 1(b), and 1(c)).

## 4. Discussion

In the present study, the tensile strength, stiffness, and antibacterial activity were significantly higher in the SNP modified L-PRF membranes than the L-PRF group. The membrane's mechanical properties were improved as a result of adding SNPs to the L-PRF and its array in the fibrin matrix. The microscopic assessment of the samples showed that the SNPs were more densely mixed with the fibrin strands in the outer layers than the inner layers. Furthermore, their precipitation was patchy in the outer layer but quite homogenous in the inner layers. This may justify the improved mechanical properties of the L-PRF membrane modified by SNPs. Yet, further studies by an electron microscope are suggested to investigate more details.

The mechanical values measured in L-PRF group were in line with the study by Khorshidi et al. who reported that, among the techniques used for making high concentrations of platelets, the L-PRF technique created more favorable mechanical properties like better tensile strength. Hence, it had better clinical handling and could be a more suitable membrane for periodontal regenerative surgical procedures. [10].

The different mechanical properties observed in different methods of making PRF and L-PRF can be due to the differences in polymerization stages, as well as the different densities of fibrin matrix and different fibrinogen concentrations [10]. The tensile strength of the tissue or the material that is sutured affects the success of suturing and the clinical result of wound healing. Thus in clinical use, the membranes with higher tensile strength are more tear resistant and bear greater applied forces [8].

The membrane produced in this study offered better mechanical properties besides maintaining its molecular properties. L-PRF can be a biologic scaffold and a reservoir for the growth factors in tissue regeneration. This fibrin increases the proliferation, migration, and differentiation of human bone marrow stem cells [18]. It also causes the expression of extracellular phosphorylated protein kinase and osteoprotegerin in osteoblasts and considerably affects the bone regeneration [19].

On the other side, the most common postoperative complication of regenerative surgeries is the membrane exposure, in which case, the oral cavity microorganisms can be colonized on the membrane and jeopardize the success of treatment [12]. Certainly, it results in increased infection risk and poor bone repair. Thus, reinforcing the antimicrobial feature of membranes can contribute to the improvement of the treatment outcome.

The SNPs are highly biocompatible and have favorable properties such as antimicrobial property [13]. Despite the several proposed theories, controversy exists regarding the mechanism of action of SNPs on microbes [20]. Silver nanoparticles can adhere to the cell membrane of the bacteria and make it porous, which consequently changes the permeability of the cell membrane and causes cell death [21].

A number of studies claimed that the contact between the silver nanoparticles and bacteria forms free radicals which can damage the bacterial cell membrane and cause bacterial death through increasing the permeability [12]. It was also announced that the Ag ions released from the nanoparticles could interfere with the thiol group of enzymes and deactivate them [22].

Several studies showed that these materials can influence a wide spectrum of gram-negative and gram-positive bacteria and also antibiotic-resistant species. They have also been proven to have antifungal and antiviral effects [12]. Bone cement reinforced with nanosilver can destroy different bacteria such as* Staphylococcus epidermidis*, methicillin-resistant* Staphylococcus epidermidis*, and methicillin-resistant* Staphylococcus aureus*, in vitro [17].

Adding SNPs to oral mouthwashes considerably reduced the growth of* Streptococcus mutans* compared with antibiotics and chlorhexidine [23]. In endodontic treatments, application of different concentrations of SNPs in combination with MTA and CEM had shown an antibacterial effect against* Escherichia coli, Enterococcus faecalis, Candida albicans,* and* Actinomyces* spp. [24]. The membrane designed in the present study succeeded to offer suitable antimicrobial properties like the previously mentioned studies.

*Viridans* group* Streptococcus* (VGS) is a heterogeneous group of organisms with six main subgroups and at least 30 different strains. Functioning as the normal flora of the body or pathogens, they can cause delay or failure in the healing of the surgical site. The VGS include gram-positive catalase-negative coccus with chain morphology in microscopic evaluation [25]. They are among the species whose interspecies genetic changes result in increased antibiotic resistance [12].

*Klebsiella *is an anaerobic facultative gram-negative nonmotile bacillus, the commonest type of which is* K. pneumonia*. It inhabits the oral cavity of healthy individuals sporadically and can cause oral infections in cases such as nosocomial infection, poor oral hygiene, alcohol overuse, leukemia, weak immune system, and extensive dental caries [26].

In the current study, the membrane modified by SNPs showed no VGS growth after neither 12 nor 24 hours; however, it had no effect on* K. pneumonia*.

## 5. Conclusion

In the present study, modification of L-PRF by SNPs yielded a product which can help prevent the growth of a great family of bacteria (VGS) on the surgical sites and its consequences. This membrane not only has biological advantages, but also offers better mechanical properties including higher tensile strength, stiffness, and toughness compared with the traditional membrane.
